# (*E*)-*N*,*N*′-Bis(2,6-dimethyl­phen­yl)-*N*,*N*′-bis­(trichloro­silyl)ethyl­ene-1,2-diamine

**DOI:** 10.1107/S1600536809006011

**Published:** 2009-02-28

**Authors:** Wei Yang, Ming Zhang, Guangyan Liu, Yuqiang Ding

**Affiliations:** a School of Chemical and Material Engineering, Jiangnan University, 1800 Lihu Road, Wuxi, Jiangsu Province 214122, People’s Republic of China

## Abstract

The asymmetric unit of the title compound, C_18_H_20_Cl_6_N_2_Si_2_, contains one half of the centrosymmetric mol­ecule. The two benzene rings are perpendicular to the plane of Si–N–C=C–N–Si fragment, making a dihedral angle of 89.9 (1)°. The crystal packing exhibits short inter­molecular Cl⋯Cl contacts of 3.3119 (17) Å.

## Related literature

For the geometric parameters of related compounds, see: Haaf *et al.* (1998 ▶, 2000 ▶); Baker *et al.* (2008 ▶); Jones *et al.* (2002 ▶).
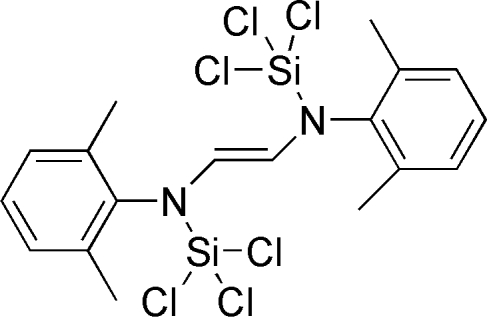


## Experimental

### 

#### Crystal data

C_18_H_20_Cl_6_N_2_Si_2_
*M* *_r_* = 533.24
Triclinic, 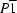

*a* = 8.1858 (3) Å
*b* = 8.4249 (3) Å
*c* = 10.6074 (4) Å
α = 74.583 (3)°
β = 79.999 (2)°
γ = 62.243 (2)°
*V* = 623.00 (4) Å^3^
*Z* = 1
Mo *K*α radiation
μ = 0.79 mm^−1^
*T* = 273 K
0.14 × 0.12 × 0.08 mm

#### Data collection

Bruker APEX2 CCD area-detector diffractometer
Absorption correction: multi-scan (*SADABS*; Bruker, 2001 ▶) *T*_min_ = 0.897, *T*_max_ = 0.939
6168 measured reflections
2168 independent reflections
1729 reflections with *I* > 2σ(*I*)
*R*_int_ = 0.034

#### Refinement

*R*[*F* ^2^ > 2σ(*F* ^2^)] = 0.059
*wR*(*F* ^2^) = 0.202
*S* = 1.11
2168 reflections
129 parameters
H-atom parameters constrained
Δρ_max_ = 0.57 e Å^−3^
Δρ_min_ = −0.50 e Å^−3^

### 

Data collection: *APEX2* (Bruker, 2005 ▶); cell refinement: *SAINT* (Bruker, 2005 ▶); data reduction: *SAINT*; program(s) used to solve structure: *SIR97* (Altomare *et al.*, 1999 ▶); program(s) used to refine structure: *SHELXL97* (Sheldrick, 2008 ▶); molecular graphics: *PLATON* (Spek, 2009 ▶); software used to prepare material for publication: *SHELXL97*.

## Supplementary Material

Crystal structure: contains datablocks global, I. DOI: 10.1107/S1600536809006011/cv2522sup1.cif

Structure factors: contains datablocks I. DOI: 10.1107/S1600536809006011/cv2522Isup2.hkl

Additional supplementary materials:  crystallographic information; 3D view; checkCIF report

## Figures and Tables

**Fig. 1.**
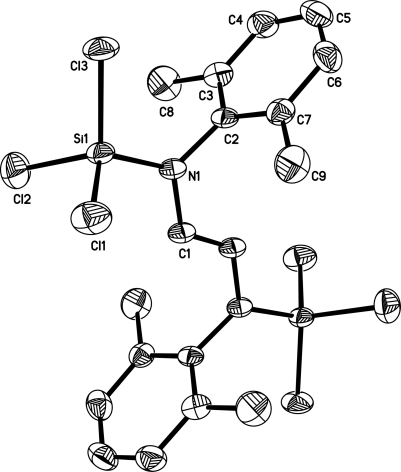
The molecular structure of (I) showing 30% probability displacement ellipsoids and the atom-numbering scheme. The unlabelled atoms are related with the labelled ones by symmetry operation (-x, -y + 2, -z). H atoms omitted for clarity.

## supplementary crystallographic information

### Comment

The title compound, (I), was synthesized by the reaction of the excess silicon tetrachloride and the dilithium salt of the diimine [N(2,6-Me_2_C_6_H_3_)C(H)]_2_ in THF (Haaf *et al.*, 1998). A high yielding preparation of the title compound was devised whereby two equivalents of SiCl_4_ were treated with dilithium salt in THF (Baker *et al.*, 2008). The title compound and related compounds are of interest in silylene chemistry in relation to synthesis of the silylene dichloride precursor (Haaf *et al.*, 2000; Baker *et al.*, 2008).

The title molecule (Fig. 1) exists in an E configuration with respect to the C═C double bond (Table 1) and crystallizes in the triclinic space group *P*1.The planes of the two xylyl substituents at the nitrogen atoms are perpendicularly oriented to the plane of Si1/N1/C1/C1^i^/N1^i^/Si1^i^ [symmery code: (i) -x, 2-y, -z] forming dihedral angles of 89.9 (1)°. The Si–Cl and Si–N bond lengths  in (I) (Table 1) are slightly shorter than those in the related complex (C_5_H_3_N-6-Me-2-NSiMe_3_)SiCl_3_ [Si–Cl 2.058 (2)-2.107 (3) Å; Si–N 1.753 (5) Å) (Jones *et al.*, 2002). The distance N1–C1[1.428 (4) Å] agrees well with that observed in the related *E*-ethenediamine complex (Baker *et al.*, 2008). The C1–N1–C2 angle [118.7 (3) °] in (I) is comparable to that in [PhC(NtBu)_2_]SiCl [120.70 (11) °] (Haaf *et al.*, 1998). The C1–N1–Si1 angle [120.1 (2) °] in (I) is larger than that in [Si[N(tBu)CH]_2_]_2_ [109.42 (14) °] (Haaf *et al.*, 1998), because of E configuration.

The crystal packing exhibits short intermolecular Cl···Cl contacts (Table 1).

### Experimental

All manipulations were carried out under an argon atmosphere using standard Schlenk techniques or a nitrogen-filled glovebox. Solvents (THF, toluene) were dried over sodium and freshly distilled prior to use.

Naphthalene (1.24 g,10 mmol) was dissolved in THF (15 ml) and lithium powder (71 mg, 10 mmol) added. The resultant suspension was stirred at room temperature for 4 h to give an green suspension. [N(2,6-Me_2_C_6_H_3_)C(H)]_2_ (1.17 g, 4.5 mmol) was added to the suspension after cooled to -78 *^o^*C. The resultant mixture was stirred at room temperature for 24 h to give a red solution. At -78 *^o^*C, silicon tetrachloride (10 ml, 88 mmol) was added to the solution. Warmed to room temperature and stirred for 24 h. Volatiles were removed in vacuo and the residue was extracted with toluene (20 ml). After filtration, the filtrate was placed at -30 *^o^*C to give yellow crystals (43%). Elemental analysis(%) calcd. for C_18_H_20_Cl_6_N_2_Si_2_: C, 40.54%; H, 3.78%; N, 5.25%; Found: C,40.61%; H, 3.83%; N, 5.17%.

### Refinement

All H atoms were placed in geometrically idealized positions and constrained to ride on their parent atoms with C–H of 0.93-0.96 Å, and *U*_iso_(H) = 1.2-1.5 U_eq_ (C).

### Crystal data

**Table:**

C_18_H_20_Cl_6_N_2_Si_2_	*Z* = 1
*M**_r_* = 533.24	*F*(000) = 272
Triclinic, *P*1	*D*_x_ = 1.421 Mg m^−^^3^
Hall symbol: -P 1	Mo *K*α radiation, λ = 0.71073 Å
*a* = 8.1858 (3) Å	Cell parameters from 2127 reflections
*b* = 8.4249 (3) Å	θ = 2.8–25.6°
*c* = 10.6074 (4) Å	µ = 0.79 mm^−^^1^
α = 74.583 (3)°	*T* = 273 K
β = 79.999 (2)°	Block, yellow
γ = 62.243 (2)°	0.14 × 0.12 × 0.08 mm
*V* = 623.00 (4) Å^3^	

### Data collection

**Table:**

Bruker APEX2 CCD area-detector diffractometer	2168 independent reflections
Radiation source: fine-focus sealed tube	1729 reflections with *I* > 2σ(*I*)
graphite	*R*_int_ = 0.034
φ and ω scans	θ_max_ = 25.1°, θ_min_ = 2.0°
Absorption correction: multi-scan (*SADABS*; Bruker, 2001)	*h* = −9→9
*T*_min_ = 0.897, *T*_max_ = 0.939	*k* = −10→9
6168 measured reflections	*l* = −12→12

### Refinement

**Table:**

Refinement on *F*^2^	0 restraints
Least-squares matrix: full	H-atom parameters constrained
*R*[*F*^2^ > 2σ(*F*^2^)] = 0.059	*w* = 1/[σ^2^(*F*_o_^2^) + (0.133*P*)^2^ + 0.1233*P*] where *P* = (*F*_o_^2^ + 2*F*_c_^2^)/3
*wR*(*F*^2^) = 0.202	(Δ/σ)_max_ = 0.088
*S* = 1.11	Δρ_max_ = 0.57 e Å^−^^3^
2168 reflections	Δρ_min_ = −0.50 e Å^−^^3^
129 parameters	

### Special details

**Table:**

Geometry. All e.s.d.'s (except the e.s.d. in the dihedral angle between two l.s. planes) are estimated using the full covariance matrix. The cell e.s.d.'s are taken into account individually in the estimation of e.s.d.'s in distances, angles and torsion angles; correlations between e.s.d.'s in cell parameters are only used when they are defined by crystal symmetry. An approximate (isotropic) treatment of cell e.s.d.'s is used for estimating e.s.d.'s involving l.s. planes.
Refinement. Refinement of *F*^2^ against ALL reflections. The weighted *R*-factor *wR* and goodness of fit *S* are based on *F*^2^, conventional *R*-factors *R* are based on *F*, with *F* set to zero for negative *F*^2^. The threshold expression of *F*^2^ > σ(*F*^2^) is used only for calculating *R*-factors(gt) *etc*. and is not relevant to the choice of reflections for refinement. *R*-factors based on *F*^2^ are statistically about twice as large as those based on *F*, and *R*- factors based on ALL data will be even larger.

### Fractional atomic coordinates and isotropic or equivalent isotropic displacement parameters (Å^2^)

**Table:**

	*x*	*y*	*z*	*U*_iso_*/*U*_eq_	
Si1	0.34542 (14)	0.80312 (14)	0.19720 (9)	0.0424 (4)	
Cl1	0.40381 (19)	1.01460 (17)	0.18495 (14)	0.0736 (5)	
Cl2	0.54249 (18)	0.64298 (19)	0.08286 (14)	0.0791 (5)	
Cl3	0.36689 (17)	0.65899 (17)	0.38326 (10)	0.0675 (4)	
N1	0.1308 (4)	0.8729 (4)	0.1525 (3)	0.0394 (7)	
C1	0.0820 (5)	0.9686 (5)	0.0214 (3)	0.0424 (9)	
H1	0.1729	0.9875	−0.0375	0.051*	
C2	−0.0078 (5)	0.8409 (5)	0.2452 (3)	0.0391 (8)	
C3	−0.0237 (6)	0.6787 (5)	0.2607 (4)	0.0510 (10)	
C4	−0.1436 (6)	0.6423 (6)	0.3580 (5)	0.0630 (12)	
H4	−0.1535	0.5335	0.3706	0.076*	
C5	−0.2502 (7)	0.7654 (8)	0.4378 (5)	0.0669 (13)	
H5	−0.3294	0.7380	0.5042	0.080*	
C6	−0.2389 (6)	0.9273 (7)	0.4189 (4)	0.0623 (12)	
H6	−0.3130	1.0103	0.4716	0.075*	
C7	−0.1181 (6)	0.9699 (6)	0.3218 (4)	0.0484 (9)	
C8	0.0925 (9)	0.5424 (7)	0.1755 (7)	0.0816 (16)	
H8A	0.2172	0.4774	0.2026	0.122*	
H8B	0.0917	0.6064	0.0858	0.122*	
H8C	0.0426	0.4569	0.1838	0.122*	
C9	−0.1071 (8)	1.1485 (6)	0.3024 (6)	0.0736 (14)	
H9A	−0.0312	1.1381	0.3666	0.110*	
H9B	−0.2290	1.2451	0.3122	0.110*	
H9C	−0.0541	1.1761	0.2162	0.110*	

### Atomic displacement parameters (Å^2^)

**Table:**

	*U*^11^	*U*^22^	*U*^33^	*U*^12^	*U*^13^	*U*^23^
Si1	0.0486 (7)	0.0433 (6)	0.0322 (6)	−0.0209 (5)	−0.0094 (4)	0.0021 (4)
Cl1	0.0869 (10)	0.0678 (8)	0.0823 (9)	−0.0503 (7)	−0.0180 (7)	−0.0021 (6)
Cl2	0.0655 (8)	0.0787 (9)	0.0780 (9)	−0.0180 (7)	0.0111 (6)	−0.0291 (7)
Cl3	0.0699 (8)	0.0822 (8)	0.0431 (6)	−0.0385 (7)	−0.0239 (5)	0.0208 (6)
N1	0.0483 (18)	0.0413 (16)	0.0266 (14)	−0.0197 (14)	−0.0087 (12)	−0.0002 (12)
C1	0.054 (2)	0.0413 (19)	0.0289 (18)	−0.0214 (18)	−0.0087 (15)	0.0009 (15)
C2	0.047 (2)	0.0440 (19)	0.0252 (16)	−0.0215 (17)	−0.0092 (14)	0.0006 (15)
C3	0.059 (3)	0.046 (2)	0.050 (2)	−0.025 (2)	−0.0104 (19)	−0.0050 (18)
C4	0.064 (3)	0.061 (3)	0.068 (3)	−0.038 (2)	−0.009 (2)	0.002 (2)
C5	0.061 (3)	0.094 (4)	0.047 (2)	−0.045 (3)	−0.001 (2)	0.001 (2)
C6	0.054 (3)	0.082 (3)	0.045 (2)	−0.024 (2)	0.0039 (19)	−0.021 (2)
C7	0.052 (2)	0.050 (2)	0.040 (2)	−0.0185 (19)	−0.0105 (17)	−0.0067 (17)
C8	0.096 (4)	0.059 (3)	0.100 (4)	−0.039 (3)	0.013 (3)	−0.035 (3)
C9	0.085 (4)	0.059 (3)	0.081 (3)	−0.029 (3)	0.004 (3)	−0.033 (3)

### Geometric parameters (Å, °)

**Table:**

Si1—N1	1.684 (3)	C4—H4	0.9300
Si1—Cl3	2.0119 (13)	C5—C6	1.369 (7)
Si1—Cl2	2.0142 (17)	C5—H5	0.9300
Si1—Cl1	2.0170 (14)	C6—C7	1.396 (6)
N1—C1	1.428 (4)	C6—H6	0.9300
N1—C2	1.444 (4)	C7—C9	1.507 (6)
C1—C1^i^	1.307 (8)	C8—H8A	0.9600
C1—H1	0.9300	C8—H8B	0.9600
C2—C7	1.399 (6)	C8—H8C	0.9600
C2—C3	1.397 (5)	C9—H9A	0.9600
C3—C4	1.372 (6)	C9—H9B	0.9600
C3—C8	1.509 (7)	C9—H9C	0.9600
C4—C5	1.386 (7)		
			
Cl3···Cl3^ii^	3.3119 (17)		
			
N1—Si1—Cl3	108.46 (11)	C6—C5—C4	120.1 (4)
N1—Si1—Cl2	112.31 (12)	C6—C5—H5	120.0
Cl3—Si1—Cl2	108.64 (7)	C4—C5—H5	120.0
N1—Si1—Cl1	112.55 (11)	C5—C6—C7	121.1 (4)
Cl3—Si1—Cl1	109.04 (7)	C5—C6—H6	119.4
Cl2—Si1—Cl1	105.73 (7)	C7—C6—H6	119.4
C1—N1—C2	118.7 (3)	C2—C7—C6	117.8 (4)
C1—N1—Si1	120.1 (2)	C2—C7—C9	121.8 (4)
C2—N1—Si1	121.2 (2)	C6—C7—C9	120.4 (4)
C1^i^—C1—N1	124.4 (4)	C3—C8—H8A	109.5
C1^i^—C1—H1	117.8	C3—C8—H8B	109.5
N1—C1—H1	117.8	H8A—C8—H8B	109.5
C7—C2—C3	121.4 (3)	C3—C8—H8C	109.5
C7—C2—N1	119.1 (3)	H8A—C8—H8C	109.5
C3—C2—N1	119.5 (3)	H8B—C8—H8C	109.5
C4—C3—C2	118.7 (4)	C7—C9—H9A	109.5
C4—C3—C8	120.2 (4)	C7—C9—H9B	109.5
C2—C3—C8	121.0 (4)	H9A—C9—H9B	109.5
C3—C4—C5	120.9 (4)	C7—C9—H9C	109.5
C3—C4—H4	119.6	H9A—C9—H9C	109.5
C5—C4—H4	119.5	H9B—C9—H9C	109.5
			
Cl3—Si1—N1—C1	−176.0 (2)	N1—C2—C3—C4	173.7 (4)
Cl2—Si1—N1—C1	−55.9 (3)	C7—C2—C3—C8	178.5 (4)
Cl1—Si1—N1—C1	63.3 (3)	N1—C2—C3—C8	−4.4 (6)
Cl3—Si1—N1—C2	5.2 (3)	C2—C3—C4—C5	1.4 (7)
Cl2—Si1—N1—C2	125.3 (3)	C8—C3—C4—C5	179.5 (5)
Cl1—Si1—N1—C2	−115.5 (3)	C3—C4—C5—C6	1.0 (7)
C2—N1—C1—C1^i^	0.4 (7)	C4—C5—C6—C7	−1.5 (7)
Si1—N1—C1—C1^i^	−178.4 (4)	C3—C2—C7—C6	2.9 (6)
C1—N1—C2—C7	−91.3 (4)	N1—C2—C7—C6	−174.2 (3)
Si1—N1—C2—C7	87.6 (4)	C3—C2—C7—C9	−177.7 (4)
C1—N1—C2—C3	91.6 (4)	N1—C2—C7—C9	5.2 (6)
Si1—N1—C2—C3	−89.6 (4)	C5—C6—C7—C2	−0.5 (6)
C7—C2—C3—C4	−3.4 (6)	C5—C6—C7—C9	−179.9 (5)

Symmetry codes: (i) −*x*, −*y*+2, −*z*; (ii) −*x*+1, −*y*+1, −*z*+1.
